# Enhanced cortical activity of swallowing under acid stimulation in normal individuals: an fNIRS study

**DOI:** 10.3389/fneur.2025.1542202

**Published:** 2025-04-07

**Authors:** Shuang Gong, WeiJun Sun, LingLing Wu, JiLiang Kang, Min Tang

**Affiliations:** ^1^Neurorehabilitation Department of Ningbo Rehabilitation Hospital, Ningbo, Zhejiang, China; ^2^School of Rehabilitation Medicine, Gannan Medical University, Ganzhou, Jiangxi, China

**Keywords:** functional near-infrared spectroscopy, acid stimulation, swallowing, cortical activity, oxyhemoglobin

## Abstract

**Introduction:**

The aims of this study are to investigate the activation patterns of the cerebral cortex in healthy individuals during liquid swallowing, as well as the differences in cerebral cortex activation between swallowing distilled water and swallowing acidic solutions, using functional near-infrared spectroscopy (fNIRS).

**Methods:**

Eighteen healthy right-handed volunteers participated in this study. Each volunteer randomly completed two swallowing tasks: swallowing distilled water and swallowing an acidic solution, which differed in taste. By analyzing the average concentration of oxyhemoglobin across various channels, we observed the activation patterns and differences in brain regions when volunteers performed different swallowing tasks.

**Results:**

During the act of swallowing distilled water, the significantly activated brain regions in the prefrontal cortex included the bilateral inferior frontal cortex and the right Broca’s area. When swallowing an acidic solution, the significantly activated regions in the prefrontal cortex were the bilateral inferior frontal cortex (IFC), bilateral orbitofrontal cortex (OFC), bilateral dorsolateral prefrontal cortex (DLPFC), right Broca’s area, left primary somatosensory cortex (S1), and left premotor/supplementary motor area (PMC/SMA). Paired t-tests revealed that the activation levels during the swallowing of acidic liquid were higher than those during the swallowing of distilled water in the bilateral dorsolateral prefrontal cortex, left primary somatosensory cortex, and left premotor/supplementary motor area.

**Conclusion:**

Functional near-infrared spectroscopy (fNIRS) can be applied to research on brain functions related to swallowing. It has revealed differences in the activation of brain regions between healthy individuals when swallowing distilled water and sour solutions. Swallowing sour liquids activates more brain areas compared to swallowing water, suggesting that sour stimuli effectively activate the swallowing cortical network.

## 1 Introduction

Swallowing is a complex, repetitive action accomplished through the interplay of external stimuli such as pressure, temperature, and chemicals. It involves the coordination of swallowing-related muscle groups (including the hyoid muscles, pharyngeal muscles, and upper esophageal muscles) and the regulation of relevant brain functional areas. The cortical regions contain relatively advanced swallowing centers that primarily initiate swallowing movements and control the functions of swallowing during the oral and pharyngeal phases. Additionally, these centers adjust swallowing-related patterns by modulating the subthreshold excitation of the brainstem swallowing center (1, 2).

Sensory training is a form of neuroplasticity-based therapy that can be categorized into various types based on the type of sensory stimulation, including thermal stimulation, olfactory stimulation, gustatory stimulation, vibrational stimulation, among others. By stimulating the chemical-sensing ion channels and transient receptor potential channels of neurons in the swallowing-related brain regions, it alters the permeability of ion channels and activates corresponding receptors. This, in turn, promotes neuroregulation of sensation and movement in the cerebral cortex, enhances sensory input and motor output related to swallowing function, strongly stimulates the nervous system, and improves patients’ swallowing ability (3). Acidic stimulation, a type of gustatory stimulation, is commonly used in clinical practice for the treatment of dysphagia and has become a routine and effective therapeutic approach. In brain functional imaging studies related to swallowing, electroencephalography (EEG), positron emission tomography (PET), functional magnetic resonance imaging (fMRI), and functional near-infrared spectroscopy (fNIRS) are commonly used. EEG has high temporal resolution but low spatial resolution. While PET and fMRI offer high spatial resolution, they pose a significant aspiration risk and are prone to motion artifacts during supine swallowing. fNIRS combines the advantages of the aforementioned methods. Although it cannot detect deep brain tissue, it has high temporal resolution, is relatively insensitive to motion artifacts, is cost-effective, and easy to operate. By detecting changes in hemoglobin concentration in functional brain regions during swallowing tasks, fNIRS can indirectly reflect neuronal activity, explore activated areas of the cerebral cortex during swallowing, and provide new means for studying the central mechanisms of swallowing. A sour bolus significantly increases swallowing frequency compared to water and sweet stimuli, becoming the primary factor affecting swallowing. Within 17 to 22 s after swallowing, the average hemodynamic responses evoked by different taste stimuli show significant differences, primarily due to taste. Specifically, the hemodynamic response during the swallowing of a sour bolus is markedly stronger than that of water. Additionally, the study also found a positive correlation trend between swallowing frequency and hemodynamic response in the early stages of motor areas, further emphasizing the significant impact of sour taste on swallowing activity (4). Existing fNIRS studies primarily focus on swallowing saliva and water as the main task paradigms to explore swallowing-related cerebral cortex activation patterns. Research based on oral sensory stimulation is scarce, and most swallowing task paradigms involve a single swallow of a small amount of liquid, neglecting the continuity of swallowing movements in natural conditions.

Given the above, this study aims to utilize fNIRS technology to deeply analyze the changes in cerebral hemodynamics in healthy adults when continuously performing two different tasks: swallowing distilled water and sour solutions. Specifically, we focus on the activation patterns of swallowing-related brain regions under sour stimulation, with the goal of revealing differences in hemodynamic responses between swallowing sour solutions and distilled water. We hypothesize that sour stimulation will elicit more significant hemodynamic responses. This research holds significant importance for deepening the understanding of the central therapeutic mechanisms related to sour stimulation and for guiding the rehabilitation treatment of swallowing disorders.

## 2 Materials and methods

### 2.1 Participants

**Figure 1:**
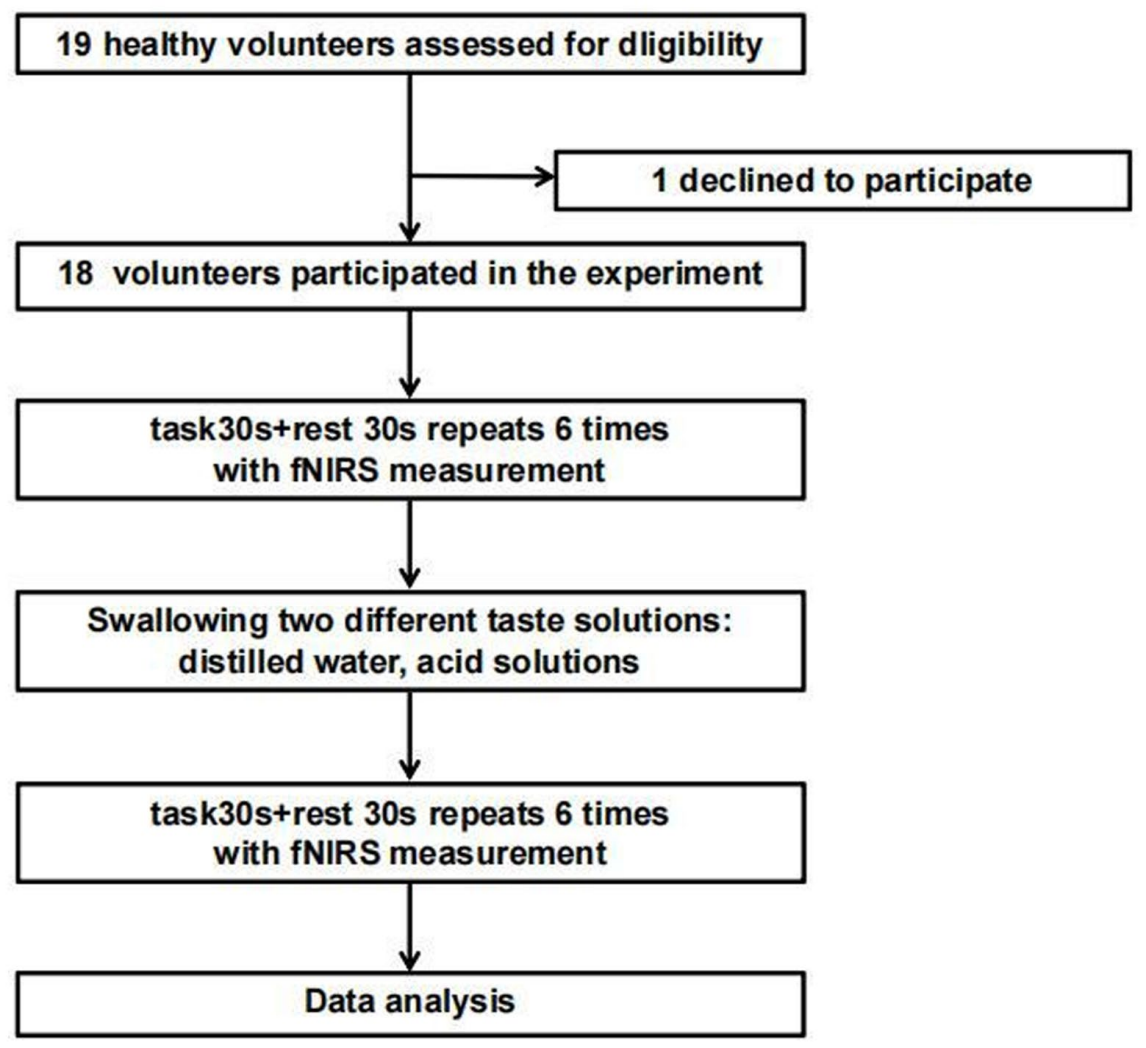
Experimental flow chart.

The study subjects were 19 right-handed healthy volunteers recruited through a WeChat group between July 2023 and March 2024. This study was approved by the Ethics Committee of Ningbo Rehabilitation Hospital (approval number: 2023024), and informed consent was obtained from all participants before their inclusion. The exclusion criteria were: ① individuals with physiological abnormalities of taste or neurological dysfunction, ② those who had participated in other experiments in the past 3 months, ③ those who had recently taken medication, ④ those with sleep disorders, and ⑤ those who had consumed coffee, alcohol, tea, or other beverages within 2 h before the experiment. The experimental flowchart is shown in Figure 1.

### 2.2 Study design

#### 2.2.1 Preparation of supplies

Disposable paper cups, disposable medical rubber examination gloves, 60 mL syringes, rubber tubing, electronic scale, distilled water, and acetic acid solution. Before the experiment, both distilled water and the acid solution were heated to the same temperature as the room temperature, with a thermostatic mat used to control the temperature of the liquids, maintaining it at 26°C. A 0.08 M acetic acid solution was prepared by mixing edible white vinegar (Zhenjiang Hennis 6° white vinegar) with distilled water.

#### 2.2.2 Swallowing task procedure

Before the experiment, the experimenters explained and demonstrated the experimental tasks to ensure that the subjects fully understood the process and avoided making any unrelated movements. After entering the room (maintained at a temperature of 25–26°C) and resting quietly for 5 min, the subjects wore near-infrared equipment and randomly completed two swallowing tasks: swallowing distilled water and an acidic solution, maintaining closed eyes throughout the experiment. The experimenters followed the prompts on the computer screen to complete the experimental procedure.

**Figure 2:**
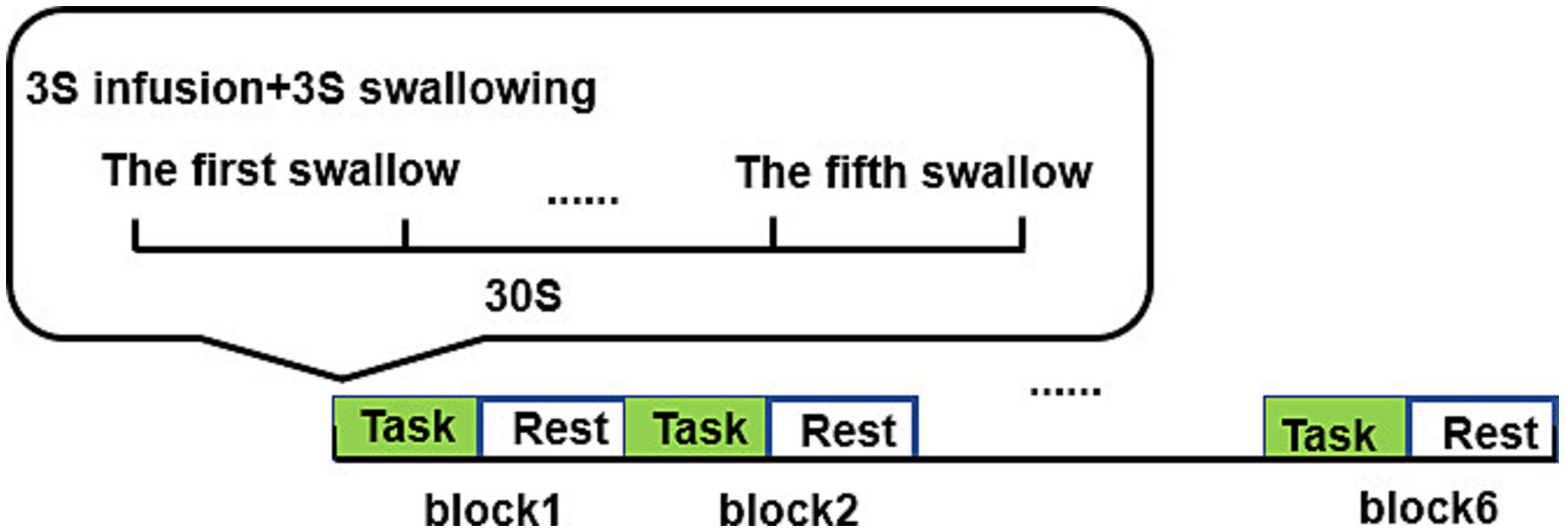
Swallowing task paradigm.

Both swallowing tasks for the two different tastes followed the same paradigm, consisting of six blocks, with each block including a 30-s task and a 30-s rest (Resting state), totaling 6 min. Each task block involved a 3-s injection and a 3-s swallowing action, with a total of five swallows of liquid. One end of the straw was placed in the subject’s mouth and fixed with medical adhesive tape, while the other end was connected to a syringe. When the screen background turned black and started counting down, the experimenter injected 3 mL of liquid into the subject’s mouth at a constant speed. When the screen background turned white and started counting down, the subject was prompted to swallow. Only one swallowing action was allowed, and chewing was not necessary. After completing each task paradigm, the subjects rested for 10 min, and after swallowing the acidic solution, the straw was rinsed with water. The experimental procedure is shown in Figure 2.

### 2.3 Near-infrared data acquisition and processing

#### 2.3.1 Near-infrared data acquisition

**Figure 3:**
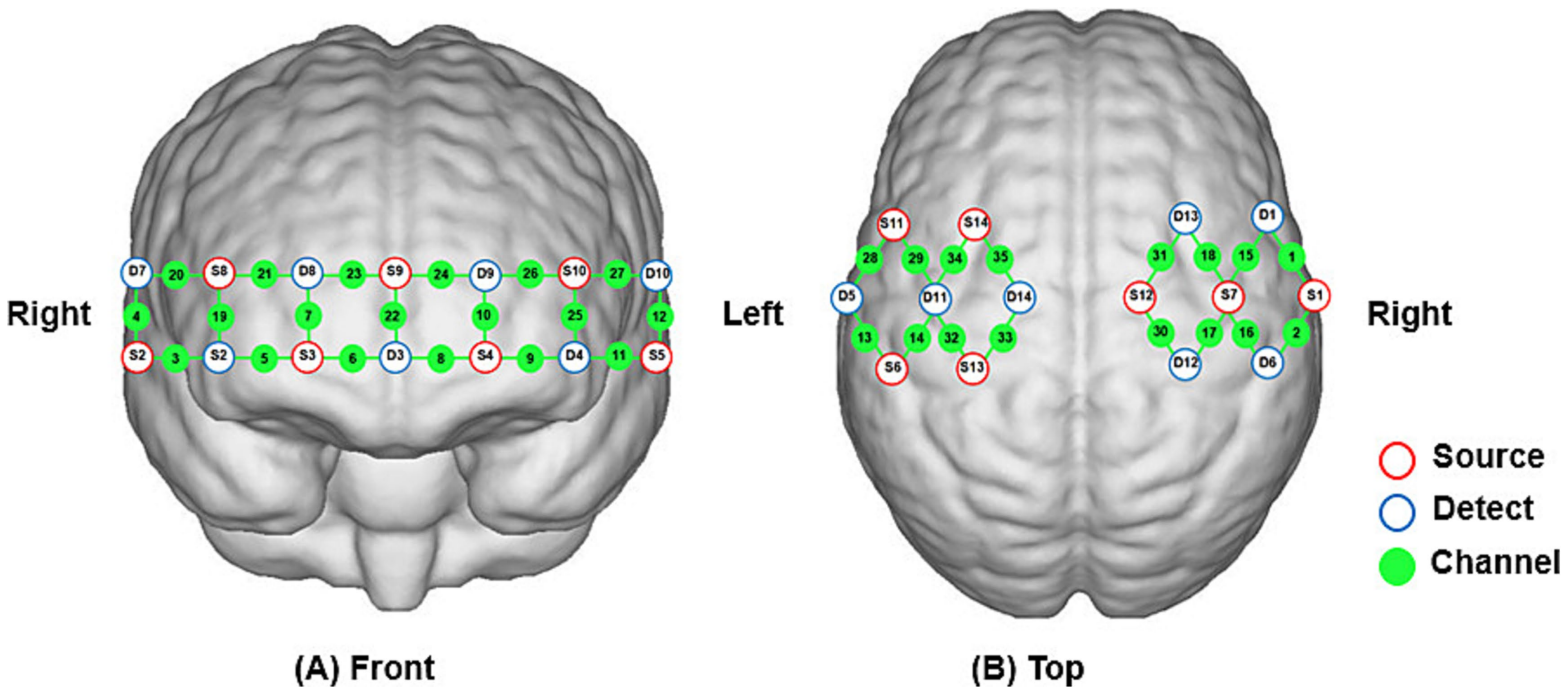
**(A,B)** Schematic diagram showing the positions of fNIRS channels and probes.

fNIRS imaging equipment (NirSmart, Hui chuang, Danyang, China) was used to acquire changes in cerebral oxyhemoglobin concentration in subjects during different task states. The equipment operates at wavelengths of 760 nm and 850 nm, with a sampling frequency of 11 Hz. A total of 35 channels were composed of 14 light source probes and 14 detector probes, with a spacing of 3 cm between each light source probe and detector probe. The probes were positioned according to the 10/20 international standard electrode system, and the fNIRS cap was placed over the bilateral prefrontal, motor, and sensory cortices of the subject. The coordinates were converted to Montreal Neurological Institute coordinates and mapped to the Montreal Neurological Institute standard brain template in NirSpace using a spatial registration technique (Danyang Hui chuang Medical Equipment Co., Ltd., China) in Figure 3.

#### 2.3.2 Data processing

The fNIRS data were processed using Nirspark 1.8.6 software.

Preprocessing: (1) Irrelevant time intervals were excluded from the experiment. (2) Motion artifacts were corrected using spline interpolation, with a standard deviation threshold of 6 and a peak threshold of 0.5. Simultaneously, the raw data were converted to optical density. (3) Filtering was applied to select components within the frequency range of 0.01–0.2 Hz to remove interference signals caused by physiological noise. (4) The differential pathlength factor (DPF) was set to 6 for both wavelengths, and based on the modified Beer–Lambert law, the filtered optical density data were calculated to obtain changes in oxyhemoglobin concentration.

#### 2.3.3 Data analysis

Block Averaging and Eigenvalue Calculation. The oxyhemoglobin (HbO_2_) concentration during the baseline interval of 2 s before the task (−2 to 0 s) was used as the baseline. After calibrating the baseline, the average amplitude of the baseline was subtracted. The six task blocks for each swallowing task under each channel were averaged after superposition to obtain the mean change in oxyhemoglobin concentration for different channels, representing cortical activation during the swallowing tasks.

### 2.4 Statistical analysis

The general information of the subjects, such as age, was expressed as mean ± standard deviation (^−^x ± s). The normality and homogeneity of variance of the measured data were tested using the Kolmogorov–Smirnov and Levene methods, respectively. A single-sample t-test was conducted to compare with the baseline values, and the mean changes in HbO_2_ concentration for the two swallowing tasks were calculated. Paired t-tests were used to analyze the mean changes in HbO_2_ between swallowing acid solution and swallowing distilled water. False Discovery Rate (FDR) correction was applied, and a *p*-value <0.05 was considered statistically significant.

### 2.5 Main observation indicator

The mean change in oxygenated hemoglobin concentration.

## 3 Results

### 3.1 fNIRS detection during tasks among subjects

One subject withdrew from the study due to intolerance to the sour-tasting liquid, while the remaining 18 subjects completed the entire experimental procedure. Among them, there were 9 males and 9 females, with a mean age of 24.81 ± 0.52 years, and all were right-handed. Among the 18 subjects, 10 subjects swallowed distilled water first, and 8 subjects swallowed acidic solution first.

### 3.2 Cortical activation during swallowing distilled water and swallowing tasks in healthy adults

**Figure 4:**
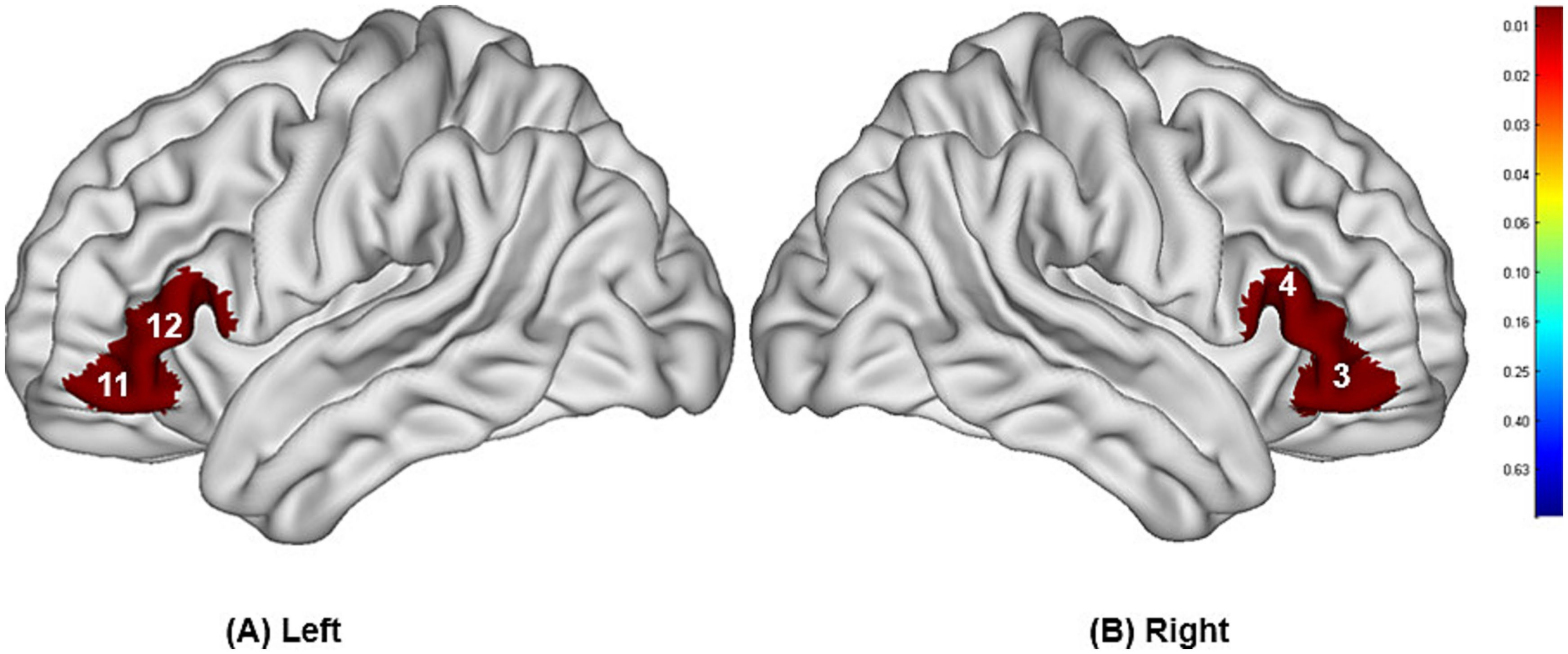
Differences in cortical activation between resting state and task state during swallowing distilled water **(A,B)**. The values displayed in the figure are *p*-values, where smaller *p*-values are represented by colors closer to red, and larger *p*-values are represented by colors closer to blue. The image only shows the regions corresponding to significantly activated channels (*p* < 0.05).

**Figure 5:**
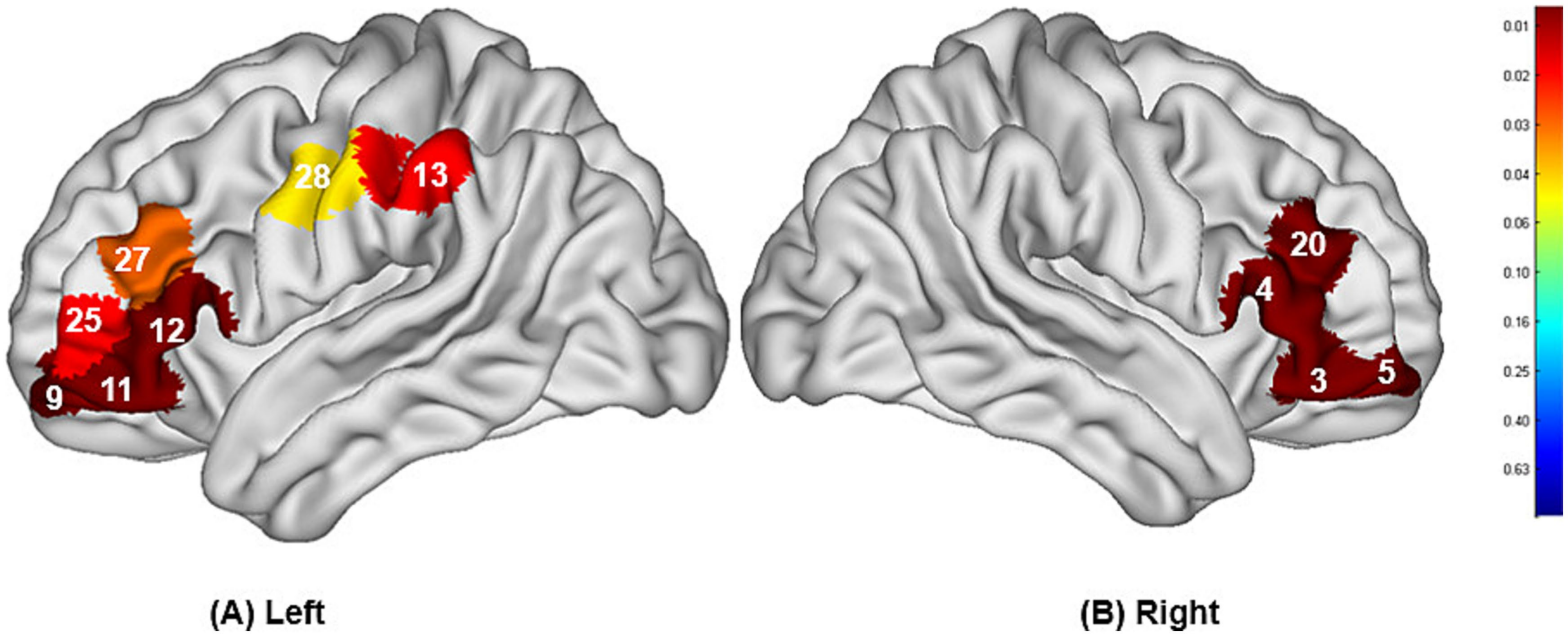
Differences in cortical activation between resting state and task state during swallowing acid solution **(A,B)**. The values displayed in the figure are *p*-values, where smaller *p*-values are represented by colors closer to red, and larger *p*-values are represented by colors closer to blue. The image only shows the regions corresponding to significantly activated channels (*p* < 0.05).

The study found that during swallowing distilled water, subjects exhibited significant activation in the bilateral IFC (channels 3, 11, 12) and the right Broca’s area (channel 4), as shown in Figure 4, with statistically significant differences (*p* < 0.05). During swallowing sour solution, significant activation was observed in the bilateral IFC (channels 3, 11, 12), bilateral OFC (channels 5, 9), bilateral DLPFC (channels 20, 27), right Broca’s area (channel 4), left S1 (channel 13), and left PMC/SMA (channel 28), as shown in Figure 5. The *p*-values and t-values for brain region activation during different tasks are presented in Tables 1, 2.

**Table 1:** The mean and *p*-value of the brain region activated when swallowing distilled water.

Channel	(S-D)	Brain area	Mean	*p*-value
3	S2-D2	R IFC	0.127 ± 0.082	0.0000658
4	S2-D7	R Broca’s area	0.149 ± 0.091	0.0000501
11	S5-D4	L IFC	0.149 ± 0.080	0.0000175
12	S5-D10	L IFC	0.134 ± 0.079	0.0000435

Values are presented as the mean ± standard deviation. *p* < 0.05 was considered statistically significant. right, R; left, L; IFC, inferior frontal cortex.

**Table 2:** The mean and *p*-value of the brain region activated when swallowing acid solution.

Channel	(S-D)	Brain area	Mean	*p*-value
3	S2-D2	R IFC	0.156 ± 0.090	0.000034
4	S2-D7	R Broca’s area	0.155 ± 0.081	0.000014
5	S3-D2	R OFC	0.061 ± 0.059	0.002740
9	S4-D4	L OFC	0.054 ± 0.049	0.001564
11	S5-D4	L IFC	0.182 ± 0.132	0.000201
12	S5-D10	L IFC	0.166 ± 0.115	0.000153
13	S6-D5	L S1	0.057 ± 0.071	0.016213
20	S8-D7	R DLPFC	0.054 ± 0.061	0.008142
25	S10-D4	L DLPFC	0.046 ± 0.057	0.016213
27	S10-D10	L DLPFC	0.059 ± 0.081	0.026881
28	S11-D5	L PMC/SMA	0.049 ± 0.075	0.047791

Values are presented as the mean ± standard deviation. *p* < 0.05 was considered statistically significant. right, R; left, L; IFC, inferior frontal cortex; OFC, orbitofrontal cortex; S1, primary somatosensory cortex; DLPFC, dorsolateral prefrontal cortex; PMC, premotor area; SMA, supplementary motor area.

### 3.3 Differences in cortical activation between swallowing distilled water and swallowing vinegar tasks in healthy adults

**Figure 6:**
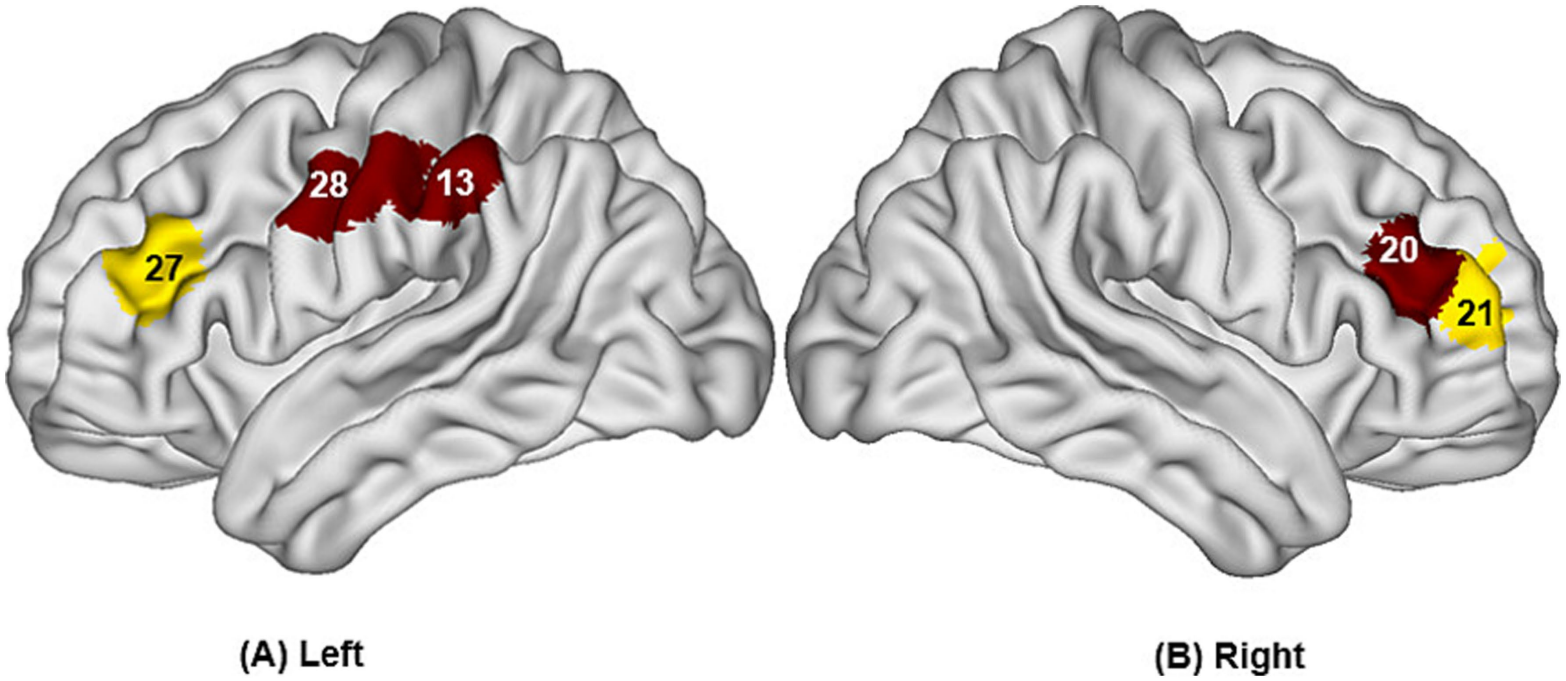
Differences in cortical activation when swallowing distilled water compared to swallowing acid solution **(A,B)**. The values displayed in the figure are *p* values, where smaller *p*-values are represented by colors closer to red, and larger *p*-values are represented by colors closer to blue. The image only shows the regions corresponding to significantly activated channels (*p* < 0.05).

Compared to the task of swallowing distilled water, there was higher activation in the bilateral OFC (channels 5, 9), bilateral DLPFC (channels 20, 27), left S1 (channel 13), and left PMC/SMA (channel 28), with statistically significant differences (*p* < 0.05). The brain region activation is illustrated in Figure 6 and Table 3.

**Table 3:** Compare the *t-*value and *p*-value when swallowing distilled water with swallowing acid solution.

Channel	(S-D)	Brain area	*t-*value	*p*-value
13	S6-D5	LS1	3.057	0.007138
20	S8-D7	R DLPFC	3.521	0.002624
21	S8-D8	R DLPFC	2.152	0.046050
27	S10-D10	L DLPFC	2.140	0.047197
28	S11-D5	L PMC/SMA	3.758	0.001566

Values are presented as the mean ± standard deviation. *p* < 0.05 was considered statistically significant. right, R; left, L; S1, primary somatosensory cortex; DLPFC, dorsolateral prefrontal cortex; PMC, premotor area; SMA, supplementary motor area.

## 4 Discussion

We employed fNIRS technology to investigate the cortical activation patterns in healthy individuals while they swallowed two types of liquids with distinct tastes: distilled water and acetic acid solution. The experimental results revealed that swallowing both water and acid solution extensively activated multiple regions in the cortex closely associated with swallowing function, including the IFC, OFC, DLPFC, Broca’s area, S1, PMC/SMA. Consistent with previous research, NIRS was first utilized in 2015 to describe cortical activation patterns during voluntary swallowing of 5 mL of water, with significant increases in oxygenated hemoglobin concentration observed in 21 out of 52 channels. The increased cortical areas encompassed the bilateral precentral gyrus, postcentral gyrus, inferior frontal gyrus, superior temporal gyrus, middle temporal gyrus, and superior marginal gyrus (5). Related fMRI studies have shown that during dry swallowing, water swallowing, and acid swallowing tasks, the bilateral precentral gyrus, postcentral gyrus, frontal cortex, and temporal cortex were most prominently activated (6). The activation patterns observed in this study demonstrate that fNIRS technology can detect cortical activation patterns during swallowing. During the swallowing process, there are complex interactions among sensory, cognitive regions, and the PMC/SMA, and the synergistic action of these regions collectively constitutes a sophisticated and precise neural regulatory network for swallowing behavior. For instance, the IFC not only processes sensory information but also integrates it with cognitive inputs from the DLPFC and motor planning signals from the PMC/SMA. This integration may occur through cortical–cortical and cortical–subcortical pathway networks, including the corticobulbar tract connecting the cerebral cortex and brainstem swallowing centers. Broca’s area, traditionally associated with speech, appears to play a role in orofacial motor control, potentially through its connections with the insula and primary somatosensory cortex. As part of the default mode network (DMN), the OFC may regulate swallowing behavior by integrating gustatory and emotional information and then transmitting these signals to motor planning regions. Unlike previous studies, no activation was observed in the primary motor area in this study, which may be due to differences in tongue movement, chewing motion, swallowing frequency, and bolus size leading to variations in oxygenated hemoglobin concentration. This study required volunteers to swallow only once each time they received 3 mL of liquid, without excessive tongue movement or chewing, thus not providing sufficient stimulus intensity to elicit hemodynamic responses in the primary motor cortex.

During tasks involving swallowing distilled water and acidic solutions, there was significant activation observed in the bilateral IFC and Broca’s area among the volunteers. The IFC, a subdivision of the prefrontal cortex, is associated with behavioral flexibility and self-control. It processes sensory information from the oral cavity, pharynx, and esophagus, decoding and integrating this information. Based on this, the IFC participates in the decision-making process of swallowing activities, determining whether to initiate a swallowing action and how to regulate the intensity and speed of swallowing (7). Additionally, studies have found that the oxygenated hemoglobin in the bilateral IFC undergoes rapid changes during the initiation phase of swallowing (8). Furthermore, the IFC has extensive connections with the brainstem swallowing center, other cortical regions, and cranial nerves related to swallowing. These connections enable the IFC to receive input from other brain areas, process this information, and transmit it to the corresponding effectors, thereby achieving precise regulation of swallowing activities.

Broca’s area is commonly associated with the production of motor speech (9), but early studies on the cortical control of swallowing have identified strong activation in Broca’s area during tasks involving swallowing and tongue movements (10). There are currently three possible explanations for this phenomenon. Firstly, studies have shown that sensory localization in the human oral cavity and pharynx projects to BA44 in the brain, suggesting that Broca’s area is also involved in the control of non-verbal orofacial somatosensorimotor functions (11). Secondly, the insular cortex, which is a deep structure located proximal to Broca’s area, has consistently been reported to be activated during swallowing in many studies. Based on this, it is speculated that the activation of Broca’s area during swallowing tasks may be due to the intense activation of the deep insular cortex (12). Additionally, the medial part of BA45 and the insular cortex are considered to be the primary gustatory cortex (13). Both swallowing water and acidic liquids constitute gustatory stimuli that can elicit hemodynamic changes.

Most importantly, swallowing acidic solutions not only activated the aforementioned regions but also showed significantly increased activation levels in the bilateral DLPFC, left S1, left PMC /SMA, compared to swallowing distilled water. This finding suggests that the stimulation of acidic solutions not only triggers the basic swallowing reflex but also elicits advanced cognitive processing of these stimuli in the brain, including gustatory perception, emotional responses, and possibly even the adjustment of swallowing strategies. The enhanced activation of these brain regions may be related to molecular mechanisms such as neurotransmitter release, ion channel activity, neuroplasticity changes, and the modulation of neural networks.

The OFC harbors the secondary taste cortex, which receives signals from the primary taste cortex and insular cortex and participates in the processing of gustatory information (14). Animal studies have also found that OFC neurons can guide the remapping of some neurons in the primary sensory cortex (15). In this study, when subjects swallowed distilled water, there was no significant increase in the concentration of oxygenated hemoglobin in the OFC; however, when subjects swallowed acidic solutions, there was significant activation in the bilateral OFC. This indicates that acidic solutions exert a greater modulatory effect on the OFC compared to aqueous solutions. Acidic stimuli transmit gustatory signals to the OFC by activating acid-sensing ion channels (ASICs) and transient receptor potential channels (TRPV1), among others. ASICs are a class of ion channels sensitive to protons (H^+^) and are widely distributed in taste buds and the central nervous system. Activation of ASICs by acidic stimuli leads to the inflow of calcium ions (Ca2^+^), which in turn triggers the release of presynaptic neurotransmitters (such as glutamate), enhancing the excitability of OFC neurons (16). Furthermore, the OFC is closely related to the dopaminergic system. Acidic stimuli may project to the OFC through midbrain dopaminergic neurons (such as the ventral tegmental area), enhancing dopamine release and thereby regulating OFC neural activity (17). The activity of the OFC is positively correlated with gustatory pleasure ratings (18). This means that if subjects find acidic solutions pleasant, the degree of OFC activation will increase (19). Notably, the OFC is a component of the default mode network (DMN), which typically shows increased activity during rest and decreased activity during active task performance. In this study, acidic solutions were easily identifiable for subjects, leading to DMN activation, which may influence and activate OFC activity. Studies on resting-state whole-brain functional network changes in post-stroke patients with dysphagia have found that alterations in intrinsic functional connectivity within the DMN are related to swallowing ability. Compared to stroke patients without dysphagia, those with dysphagia showed significantly reduced DMN functional connectivity. The authors speculate that acidic stimuli can affect DMN circuits, which may help identify potential therapeutic targets for dysphagia (20). In summary, the bilateral OFC plays a crucial role in swallowing control and participates in the processing of gustatory information. When subjects taste acidic solutions, the degree of OFC activation may be related to gustatory pleasure and the activation state of the DMN.

In the complex process of swallowing, the oral preparatory phase is a crucial stage primarily consisting of neuropsychological processes involving both sensory and cognitive participation, also known as the “cognitive phase” (21). This phase involves the recognition and evaluation of food or liquid, as well as the initial formation of swallowing decisions. The DLPFC is closely related to cognitive behavior (22). Acidic stimuli can enhance DLPFC activation, potentially involving the modulation of multiple neurotransmitters. Firstly, glutamate enhances the excitability of DLPFC neurons through synaptic transmission mediated by *α*-amino-3-hydroxy-5-methyl-4-isoxazolepropionic acid (AMPA) and N-methyl-D-aspartate (NMDA) receptors, thereby improving cognitive control ability (23). Secondly, acidic stimuli may also enhance the release of norepinephrine through norepinephrine fibers projecting from the locus coeruleus to the DLPFC, further promoting the cognitive functions of the DLPFC (24). Meanwhile, the DLPFC plays a key role in the preparation and planning of voluntary movements. It receives input signals from regions such as the cingulate gyrus and transmits this information to the premotor cortex and supplementary motor cortex to facilitate the execution of a series of swallowing actions (25). The research conducted by Kawai et al. also supports this notion, as they propose that the bilateral premotor cortex and supplementary motor cortex receive input signals from the cingulate gyrus and dorsolateral prefrontal cortex, working in concert to coordinate swallowing movements (26). Acidic stimuli effectively act within the swallowing network by activating the DLPFC, not only enhancing cognitive control over swallowing but also facilitating the precise execution of swallowing actions. Therefore, in the treatment and rehabilitation of swallowing disorders, strategies such as acidic stimuli to activate the DLPFC can be considered to improve patients’ swallowing function.

S1 serves as a vital area for processing sensory information, responsible for receiving, integrating, and interpreting sensory inputs from various parts of the body. Precise localization information about the bolus and oral structures is crucial for coordinating jaw and tongue movements during chewing and swallowing (27). This information is processed and conveyed by the S1 area, enabling us to precisely control the chewing force of the jaw and the direction of tongue propulsion, facilitating smooth food passage into the pharynx and ensuring the accuracy and coordination of swallowing movements (28). In this study, sour taste, as a more intense sensory input compared to distilled water, was found to cause an increase in the concentration of oxygenated hemoglobin in S1. Other studies have also observed that sour taste-induced activity in S1 is higher than that induced by water stimulation, and a gradual sensitization of S1 to acidic boli with enhanced hemodynamic responses was noted over 20 swallows (29). In healthy individuals, sour taste can increase submental muscle activity, which aids in the smooth execution of swallowing actions. In patients with swallowing disorders, acidic boli can alter swallowing duration and improve swallowing safety on the Penetration-Aspiration Scale (4, 30, 31). This suggests that sour taste stimulation may have a beneficial effect on improving swallowing function. In patients with post-stroke swallowing disorders, functional connectivity between the hypothalamus and the postcentral gyrus (part of S1) is weakened, and the degree of this weakness correlates with the severity of swallowing disorders (32). These effects may be due to increased input from the superior laryngeal nerve, facial nerve, trigeminal nerve, and glossopharyngeal nerve. These nerves convey gustatory and somatosensory information to S1, enabling it to integrate and process this information. Sour taste stimulation triggers synaptic plasticity changes by activating acid-sensing ion channels (ASICs) and transient receptor potential vanilloid subtype 1 (TRPV1) channels, enhancing S1’s processing capacity for gustatory and somatosensory information (33). Additionally, S1 activity is modulated by GABAergic inhibitory neurons, and sour taste stimulation may enhance the neural encoding efficiency of S1 by regulating the activity of GABAergic neurons, enabling it to more accurately process and transmit sensory information (34).

As key nodes in the motor network, the PMC/SMA are not only involved in initiating and controlling motor behavior but are also closely related to motor planning, particularly in relation to the sequential motor planning that occurs during swallowing (35, 36). This viewpoint is supported by multiple studies, including research by Griffin et al., which found activation in the premotor cortex and supplementary motor cortex during tongue movement (25). In the present study, the significant activation of the premotor cortex and supplementary motor area under sour taste stimulation reflects enhanced planning and fine control of swallowing movements during sour taste processing, which is consistent with previous research findings (37). These regions are closely related to oral control and pharyngeal muscle movement, involving the entire process from swallowing planning to execution (27). On the other hand, the PMC/SMA primarily process continuous movements. The subjects in this study performed a continuous swallowing task with acidic solutions, which may explain this result. Sour taste stimulation has been shown to enhance the activation of the SMA / PMC, potentially related to glutamatergic signaling and dopaminergic modulation. Glutamate enhances the excitability of PMC/SMA neurons through synaptic transmission mediated by AMPA and NMDA receptors. This increased excitability facilitates the planning and execution of swallowing movements (38). Simultaneously, sour taste stimulation may enhance dopamine release through the nigrostriatal dopaminergic pathway, thereby improving the motor planning capabilities of the PMC/SMA (39).

Under sour taste stimulation, the enhanced activation of specific brain regions is primarily mediated by a series of molecular mechanisms involving neurotransmitters and neuromodulators. The perception of sour taste initiates with the detection of protons (H^+^) by acid-sensing ion channels (ASICs) and other proton-sensitive receptors on taste receptor cells. These sensory signals are then transmitted to the brain via the facial nerve, glossopharyngeal nerve, and vagus nerve, triggering enhanced activation of the gustatory cortex (including the insula and orbitofrontal cortex) and related regions. During this process, neurotransmitters such as glutamate and *γ*-aminobutyric acid (GABA) are released within the activated brain regions, further modulating neuronal excitability and thereby enhancing the overall activation levels of the DLPFC, S1, PMC/SMA. These specific brain regions form a dynamic network, where the IFC and Broca’s area may process initial sensory perceptions and decision-making processes, while the DLPFC is involved in cognitive planning and strategic adjustments. S1 ensures accurate sensory feedback, and PMC/SMA coordinates motor execution. This network may be jointly regulated by feedforward and feedback mechanisms, where sensory information from the oral cavity and pharynx is continuously integrated with motor commands to ensure smooth and efficient swallowing. The enhanced activation in response to sour taste stimulation may reflect an increased demand for sensory discrimination and motor precision, leading to greater involvement and recruitment of these brain regions.

The findings of this study not only deepen our understanding of the neural regulatory mechanisms involved in swallowing but also reveal the interactions between the gustatory, sensory, and motor systems. Future research can further explore the impact of different gustatory stimuli (such as sweet, bitter, and salty tastes) on brain activation patterns, as well as the variations in these neural responses among specific populations (such as the elderly and patients with swallowing disorders), providing a scientific basis for clinical interventions and rehabilitation treatments. This study is an exploratory trial with a small sample size, and future research needs to expand the sample size to further support these conclusions. In addition, this study only considered cortical activation, and there is insufficient evidence regarding the reconstruction of brain network connections. Subsequent studies will further explore the mechanism of action of gustatory stimuli on brain network connections to provide more detailed research results.

## 5 Conclusion

This study, utilizing fNIRS technology, revealed differences in brain regional activation between healthy individuals when swallowing distilled water and acidic solutions. It was confirmed that swallowing acidic liquids activated more brain regions than swallowing water, including the bilateral dorsolateral prefrontal cortex, the left primary somatosensory cortex, and the left premotor/supplementary motor area. These findings suggest that sour taste stimulation can effectively activate the swallowing cortical network, providing a new perspective for understanding the neuroregulatory mechanisms involved in the swallowing process.

## Data Availability

The raw data supporting the conclusions of this article will be made available by the authors, without undue reservation.
